# Immune-related toxicity and soluble profile in patients affected by solid tumors: a network approach

**DOI:** 10.1007/s00262-023-03384-9

**Published:** 2023-03-03

**Authors:** Andrea Botticelli, Alessio Cirillo, Giulia Pomati, Enrico Cortesi, Ernesto Rossi, Giovanni Schinzari, Giampaolo Tortora, Silverio Tomao, Giulia Fiscon, Lorenzo Farina, Simone Scagnoli, Simona Pisegna, Fabio Ciurluini, Antonella Chiavassa, Sasan Amirhassankhani, Fulvia Ceccarelli, Fabrizio Conti, Alessandra Di Filippo, Ilaria Grazia Zizzari, Chiara Napoletano, Aurelia Rughetti, Marianna Nuti, Silvia Mezi, Paolo Marchetti

**Affiliations:** 1 Department of Radiological, Oncological and Pathological Science, Sapienza University of Rome, 00185 Rome, Italy; 2 Department of Molecular Medicine, Sapienza University of Rome, Viale Regina Elena 291, 00161 Rome, Italy; 3 Medical Oncology, Fondazione Policlinico Universitario Agostino Gemelli IRCCS, 00168 Rome, Italy; 4 Medical Oncology, Università Cattolica del Sacro Cuore, 00168 Rome, Italy; 5 Department of Computer, Control, and Management Engineering “Antonio Ruberti”, Sapienza University of Rome, Via Ariosto 25, 00185 Rome, Italy; 6 Department of Medical and Surgical Sciences and Translational Medicine, University of Rome “Sapienza”, 00185 Rome, Italy; 7 Guy’s and St Thomas’ NHS Foundation Trust, Westminster Bridge Rd, Bishop’s, London, SE1 7EH UK; 8 Arthritis Center, Dipartimento Di Scienze Cliniche Internistiche, Anestesiologiche E Cardiovascolari, Sapienza University of Rome, Viale del Policlinico 155, 00161 Rome, Italy; 9 Laboratory of Tumor Immunology and Cell Therapy, Department of Experimental Medicine, Policlinico Umberto I, University of Rome “Sapienza”, 00161 Rome, Italy; 10 Istituto Dermopatico Dell’Immacolata, 00167 Rome, Italy

**Keywords:** Immunotherapy, Toxicity, Soluble profile, Solid tumors, Network

## Abstract

**Background:**

Immune checkpoint inhibitors (ICIs) have particular, immune-related adverse events (irAEs), as a consequence of interfering with self-tolerance mechanisms. The incidence of irAEs varies depending on ICI class, administered dose and treatment schedule. The aim of this study was to define a baseline (T0) immune profile (IP) predictive of irAE development.

**Methods:**

A prospective, multicenter study evaluating the immune profile (IP) of 79 patients with advanced cancer and treated with anti-programmed cell death protein 1 (anti-PD-1) drugs as a first- or second-line setting was performed. The results were then correlated with irAEs onset. The IP was studied by means of multiplex assay, evaluating circulating concentration of 12 cytokines, 5 chemokines, 13 soluble immune checkpoints and 3 adhesion molecules. Indoleamine 2, 3-dioxygenase (IDO) activity was measured through a modified liquid chromatography–tandem mass spectrometry using the high-performance liquid chromatography-mass spectrometry (HPLC–MS/MS) method. A connectivity heatmap was obtained by calculating Spearman correlation coefficients. Two different networks of connectivity were constructed, based on the toxicity profile.

**Results:**

Toxicity was predominantly of low/moderate grade. High-grade irAEs were relatively rare, while cumulative toxicity was high (35%). Positive and statistically significant correlations between the cumulative toxicity and IP10 and IL8, sLAG3, sPD-L2, sHVEM, sCD137, sCD27 and sICAM-1 serum concentration were found. Moreover, patients who experienced irAEs had a markedly different connectivity pattern, characterized by disruption of most of the paired connections between cytokines, chemokines and connections of sCD137, sCD27 and sCD28, while sPDL-2 pair-wise connectivity values seemed to be intensified. Network connectivity analysis identified a total of 187 statistically significant interactions in patients without toxicity and a total of 126 statistically significant interactions in patients with toxicity. Ninety-eight interactions were common to both networks, while 29 were specifically observed in patients who experienced toxicity.

**Conclusions:**

A particular, common pattern of immune dysregulation was defined in patients developing irAEs. This immune serological profile, if confirmed in a larger patient population, could lead to the design of a personalized therapeutic strategy in order to prevent, monitor and treat irAEs at an early stage.

**Supplementary Information:**

The online version contains supplementary material available at 10.1007/s00262-023-03384-9.

## Introduction

Over the past decade immunotherapy revolutionized the field of cancer treatment by demonstrating a significant therapeutic efficacy in many solid cancers, including non-small cell lung cancer (NSCLC) [1, 2], uveal metastatic melanoma (UM) [3–5], recurrent/metastatic squamous cell carcinoma of the head and neck (R/M HNSCC) [6, 7] and renal cell carcinoma (RCC) [8, 9].

Immune checkpoint inhibitors (ICIs) are a class of immunotherapy drugs that act by blocking inhibitory signaling pathways in the immune system [10, 11]. The immune response to cancer is activated by ICIs, so that T cells could recognize and attack cancer cells, overcoming the suppression which promotes immune exhaustion and tumor escape [12–14].

ICIs are usually better tolerated than both cytotoxic chemotherapy and target therapy; nevertheless, they have a particular immune-related toxicity profile due to their mechanism of action [15, 16]. Immune-related adverse events (irAEs) may potentially affect any organ or bodily system, as they are due to the action of immune system cells on healthy tissues, interfering with self-tolerance [17, 18]. Although most of toxicity events are mild and reversible, some of them may be associated with life-threatening deterioration of organ function and decreased quality of life (QoL). This may cause temporary or permanent discontinuation of immunotherapy; in some rare cases, they could even lead to permanent damage or be fatal [19, 20]. Therefore, clinicians are facing the urgent need for identifying predictive biomarkers of immunotherapy-related toxicities, which have been poorly investigated so far.

Several predictive factors are patient-related, such as the presence of sarcopenia at baseline or low muscle attenuation (qualitative muscle reduction), both recently associated with a higher risk of developing severe treatment-related toxicity [21, 22]. Other potential underlying risk factors including a family history of autoimmune diseases and concomitant use of drugs with known immune-related toxicity such as antiarrhythmics, antibiotics, anticonvulsants or antipsychotics have been proposed for severe irAEs [23–29]. Furthermore, specific irAEs seem to be related to specific types of cancer. For example, NSCLC is associated with an earlier onset of irAEs and to an increased occurrence of interstitial lung disease, compared to melanoma [30, 31].

In addition, several circulating molecules have been studied as potential biomarkers of irAEs. A subgroup of cytokines (GM-CSF, IFN-*α*2, IL-12p70, IL-1*α*, IL-1*β*, IL-1RA, IL-2 and IL-13) [32, 33] is differently expressed in the serum of patients who develop severe irAE, both before and during ICI treatment, representing a therapeutic target in order to decrease ICI-induced irAE rates [34–36]. Cytokine inhibitors targeting TNF-*α* (infliximab, adalimumab, etanercept), IL-6 (tocilizumab) and IL-17A (secukinumab) are indicated for treating severe irAE refractory to corticosteroids [37–40].

Similarly, the soluble forms of immune checkpoints (sICs), which are either shed or released in association with microvesicles appear to be associated with irAE development in cancer patients. For example, it has been demonstrated that high levels of sCTLA4 in melanoma patients at baseline resulted in an increased risk of irAE [41]. These soluble immune-related molecules have been widely studied as possible biomarkers associated with response or survival [42–44], but their role in predicting immunotherapy-related toxicity has been considerably less investigated.

The main challenge was to identify an immune profile predictive of immune-related toxicity development at baseline. Therefore, this study evaluated a large spectrum of circulating molecules in serum of cancer patients prior to start anti-PD-1 treatment including cytokines, chemokines, soluble immune checkpoints, molecules of adhesion and indoleamine-2,3-dioxygenase (IDO) activity. The purpose was to define the immune profile of patients who would develop immune-related toxicities and to evaluate, through a network analysis, any difference in comparison with patients who would not develop toxicity during treatment.

## Materials and methods

### Patients’ enrollment and samples collection

From April 2020 to May 2021, 79 patients with NSCLC, UM, R/M HNSCC and RCC, who received immunotherapy, with the anti-PD-1 nivolumab or pembrolizumab, were enrolled in this multicentric prospective study. ICI treatment was administered intravenously as a first- or second-line setting, according to an approved schedule, until either disease progression or development of unacceptable toxicity occurred. Patient characteristics were recorded and the baseline clinical conditions were defined by means of Eastern Cooperative Oncology Group (ECOG) Performance Status (PS). Patients were clinically staged with contrast enhanced computed tomography (CT) scan and, if clinically indicated, with magnetic resonance imaging (MRI) and CT/PET at baseline (T0) and every 3 cycles of therapy.

Blood samples were collected into BD Vacutainer Plus Plastic Serum tubes (Becton Dickinson, NJ, USA) and processed within 1 h of collection. The tubes were then centrifuged at 1800 rpm for 10 min. Serum was collected and stored at − 80 °C until use. All samples were collected at T0, before the start of anti-PD1 treatment.

### Toxicities

Patients were clinically evaluated with each administration of the drug. Immune-related toxicity was identified through the performance of blood tests and clinical assessment. Toxicities/adverse effects (AEs) were recorded at day 1 of every treatment cycle and classified according to the National Cancer Institute Common Terminology Criteria for Adverse Events (version 4.0).

Based on the relevant clinical impact of the onset of adverse events, and since these require a different treatment strategy based on their severity, irAEs have been distinguished into low grade (G0-G1) and high grade (G2-G3). Cumulative toxicity defined as the presence of more than one irAE of any grade was recorded. For each patient, irAEs were treated in each patient through a multidisciplinary approach involving endocrinologists, rheumatologists, nephrologists and dermatologists, as suggested [45, 46].

### Serological evaluation of immune-related molecules

Serum collected at baseline (T0) was assayed to detect the concentration of 12 cytokines, 5 chemokines, 13 soluble immune checkpoints and 3 adhesion molecules and to evaluate IDO activity. Levels of soluble immune-related molecules were quantified using the ProcartaPlex Human Inflammation Panel (20 Plex, catalog number EPX200-12,185–901; sE-Selectin; GM-CSF; ICAM-1/CD54; IFN alpha; IFN gamma; IL-1 alpha; IL-1 beta; IL-4; IL-6; IL-8; IL-10; IL-12p70; IL-13; IL-17A/CTLA-8; IP-10/CXCL10; MCP-1/CCL2; MIP-1alpha/CCL3; MIP-1 beta/CCL4; sP-Selectin; TNF alpha) (eBioscience, Vienna, Austria) and the Human Immuno-Oncology Checkpoint 14-Plex ProcartaPlex Panel 1 (catalog number EPX14A-15803–901; BTLA; GITR; HVEM; IDO; LAG-3: 47; PD1; PD-L1; PD-L2; TIM-3; CD28; CD80; CD137; CD27; CD152) (eBioscience) according to manufacturer instructions. Samples were measured using Luminex 200 platform (BioPlex, Bio-Rad), and data, expressed in pg/ml of protein, were analyzed using Bio-Plex Manager Software. To evaluate IDO activity, serum levels of tryptophan (trp) and kynurenine (kyn) were measured through modified liquid chromatography–tandem mass spectrometry (LC–MS/MS) method. Data acquired were expressed as kyn/trp ratio. Subsequently to the evaluation the soluble molecule IDO was excluded from the analysis due to poor reliability of the multiplex method. To assess its activity is preferentially performed the LC–MS/MS method. The final version of the protocol was approved by the Institutional Ethics Committee (Ethical Committee n. n.4181, “Sapienza University of Rome”).

### Statistical analysis

A total of 34 soluble molecules from 79 patients were analyzed according to the presence of cumulative toxicities. Data were first preprocessed by applying a logarithmic transformation and normality was assessed with Shapiro–Wilk test. Soluble molecules expression levels were then test for differences between patients with or without toxicity (i.e., showing cumulative tested for significance with the Mann–Whitney test). *P* values were adjusted for multiple comparisons by applying the false discovery rate (FDR) correction [47]. Adjusted *p* values of 0.05 or less were considered to be statistically significant.

### Connectivity analysis

Biological systems respond to multiple inputs that can vary and interact simultaneously—i.e., these are complex systems that form molecular networks. Given that a gene or gene product does not exert its effect on phenotype in isolation, investigating the molecular context (i.e., the network of the functional and molecular interactions within a cell) is essential for understanding the true bases for phenotype and patho-phenotype [48]. To construct these networks, we used a quantitative approach based on the co-expression between molecules, quantifying the relationship between two molecules (connectivity) via the correlation between their expression profiles. Even if correlation does not imply causation, molecules that are co-expressed can be functionally coordinated in response to an external stimulus, implying a common way of functioning or the influence by a shared underlying mechanism [49].

In this study, we aimed to examine how the connectivity between the soluble molecules can change in patients with and without toxicity. Firstly, in order to investigate the relationships between the soluble molecules and the toxicity distributions, a connectivity heatmap was obtained by calculating Spearman correlation coefficients among each pair of soluble molecules and the cumulative toxicity values for all the patients analyzed.

Then, in order to study the differences in terms of connectivity of soluble molecules in patients with toxicity compared to patients without toxicity, two connectivity heatmaps were built by calculating the Spearman correlation coefficients among each pair of soluble molecules, one for patients with toxicity (i.e., cumulative toxicity equal to 1) and one for patients without toxicity (i.e., cumulative toxicity equal to 0). *P* values were adjusted for multiple comparisons by applying the false discovery rate (FDR) correction [47]. Adjusted *p* values of 0.05 or less were considered to be statistically significant. Specifically, by diving the cohort of subjects in patients with toxicity and without toxicity, for each group, we computed the pair-wise correlations between the soluble molecules’ expression levels, and we rendered them as a map, in which each cell reported the obtained statistically significant correlation value for each pair of molecules, encoded as a color that increases from red (negative correlation) to blue (positive correlation).

### Connectivity networks

Two different networks of connectivity were constructed, one for patients without toxicity (i.e., cumulative toxicity equal to 0) and one for patients with toxicity (i.e., cumulative toxicity equal to 1) using Spearman correlation coefficients, so that the elements of the resulting connectivity matrix were in the [− 1, 1] interval [50]. In the two networks, nodes represent soluble molecules and a link occurs between two nodes if the absolute value of Spearman correlation between their expression levels is both greater than a selected threshold (i.e., the 85th percentile of the overall distribution corresponding to 0.6) and statistically significant (adjusted *p* value ≤ 0.05). From these two networks, biomarker connection pairs which in common between patients with and without toxicity were extracted, as well as toxicity-specific ones. All the connectivity networks along with their corresponding values of correlation and statistical adjusted *p* values were detailed as edge lists in Supplementary Table 2.

## Results

### Patients

Seventy-nine metastatic patients treated with ICIs were enrolled in this study. The baseline clinical characteristics are reported in Table 1. Fifty-two (66%) patients were male, and 27 (34%) were female, with a median age of 71 years (range 50–89). The tumor type was NSCLC, UM, locally incurable R/M HNSCC and RCC in 36 (46%), 20 (25%), 13 (17%) and 10 (12%) of patients, respectively. Fifty-nine (74%) patients were treated with nivolumab and 20 (26%) with pembrolizumab. Immunotherapy was planned as a first-line treatment in 27 (33%) patients, while 42 (54%) patients were previously treated with chemotherapy line and 10 (13%) underwent targeted therapy as a first-line treatment. Forty (51%) patients developed immune-related adverse events; in particular 52 (66%) patients reported G0-G1 toxicity and 27 (34%) patients reported G2-G3 toxicity. No immune-related deaths as well as any unexpected toxicity were recorded.

**Table 1:** Baseline clinical and pathological characteristics of patients and soluble analyzed molecules

Characteristics	Patients (*N*)	(%)
*Age*		
Median Range	71 50–89	
*Gender*		
Male	52	66%
Female	27	34%
*Cancer type*		
NSCLC	36	46%
UM	20	25%
R/M HNSCC	13	17%
RCC	10	12%
*Previous treatment*		
No treatment	27	33%
Chemotherapy	42	54%
Target therapy	10	13%
*Immunotherapy*		
nivolumab	59	74
pembrolizumab	20	26
*Line of ICI treatment*		
First line	27	35%
Second line or more	52	65%
*Toxicities*		
Toxicity G0-G1	52	66%
Toxicity G2-G3	27	34%
Cumulative toxicities	27	35%
Asthenia	26	33%
Skin toxicity	17	22%
Endocrine toxicity	16	21%
Diarrhea	4	5%
Neurological symptoms	4	5%
Interstitial pneumonia	1	1%
*Soluble molecules*		
Cytokines	TNF*α*, IFN*α*, IFN*γ*, IL1*α*, IL1*β*, IL10, IL12p70, IL13, IL17A, IL4, IL6, GM-CSF	
Chemokine	MCP1, MIP-1*α*, MIP-1*β*, IL8, IP10	
Soluble immuno-checkpoint	BTLA, sCD137 sCD27, sCD28, sCD80, sCTLA-4, sGITR, HEVM, sLAG3, sPD1, sPDL-1, sPDL-2, sTIM3	
Adhesion molecules	sE-selectin, sP-selectin, sICAM-1	
IDO		

The most common toxicities recorded were asthenia, skin toxicity, endocrine toxicity, diarrhea, neurological symptoms and interstitial pneumonia in 26 (33%), 17 (22%) 16 (21%), 4 (5%), 4 (5%) patients and one (1%) patient, respectively. In addition, 27 (35%) patients developed more than one toxicity, reporting the presence of at least two irAEs (Table 1).

### Connectivity pattern correlation with toxicity

**Fig. 1:**
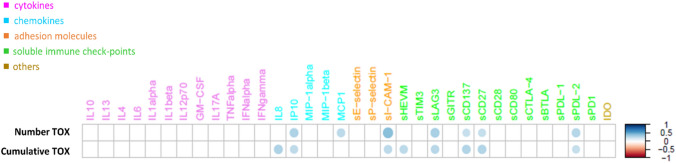
Connectivity heatmap between the soluble molecule expression profiles and the toxicity values. Statistically significant Spearman correlations (adjusted *p* value ≤ 0.05) are reported. In the plot, circles are scaled and colored according to the correlation values, increasing from red (negative correlation) to blue (positive correlation). Soluble molecules are grouped and ordered according to the functional group reported in the legend

**Fig. 2:**
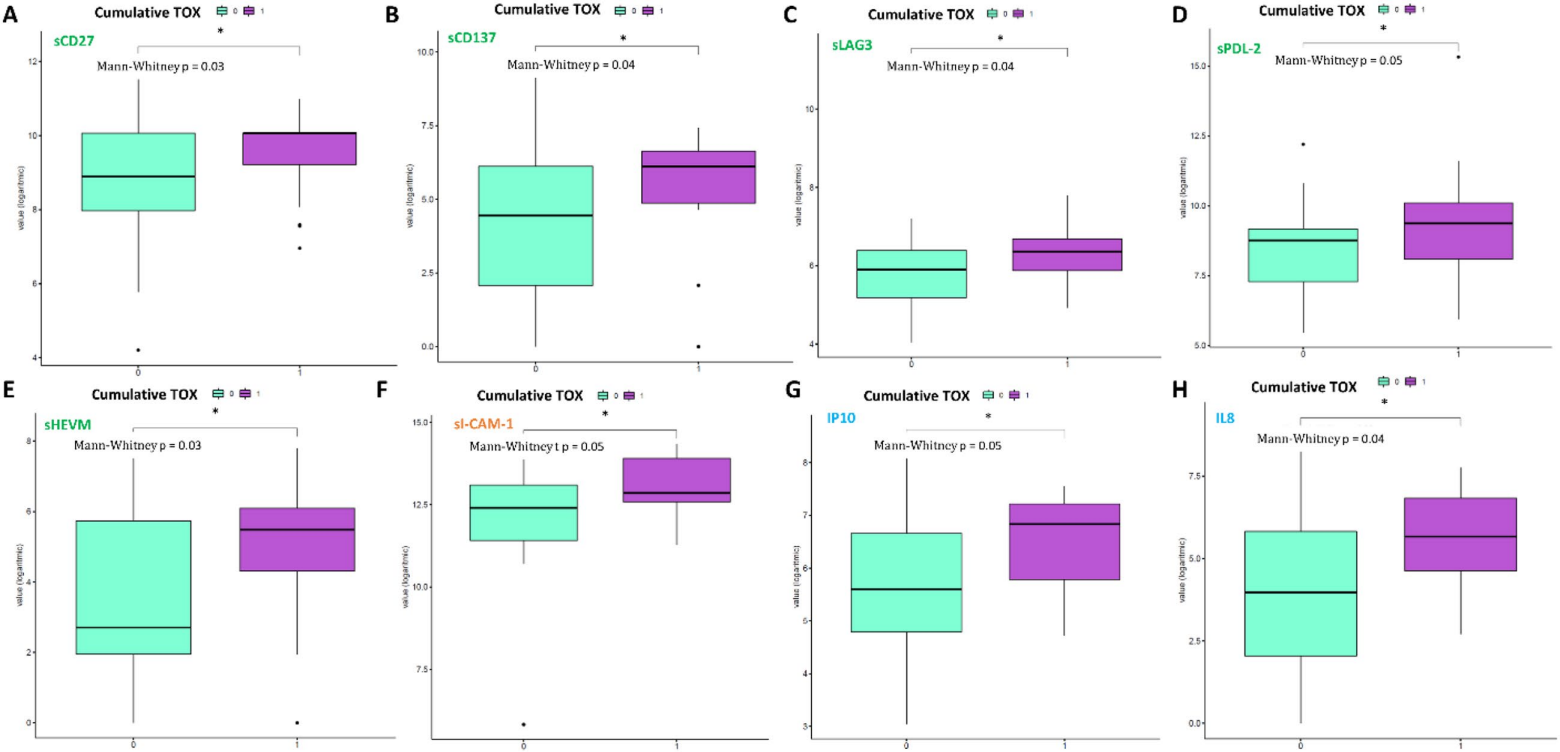
Boxplot of biomarker expression level (logarithmic scale) in 52 patients with toxicity equal to 0 (violet box) and 27 patients with toxicity equal to 1 (water blue box). Adjusted *p* values (p) were obtained by performing the Mann–Whitney test for unpaired samples. Only biomarkers showing a statistically significant difference between the two groups are reported. (* adjusted *p* value ≤ 0.05)

The correlation analysis and the computed Spearman correlation between the expression profiles of the circulating molecules under examination and the distribution of their corresponding toxicity values unveiled a positive and statistically significant (adjusted *p* value ≤ 0.05) correlation between the cumulative toxicity and a group of soluble molecules, including pro-inflammatory chemokines (i.e., IP10 and IL8), soluble immune checkpoints (i.e., sLAG3, sPDL-2, sHVEM, sCD137, sCD27) and one soluble adhesion molecule (i.e., sICAM-1) (Fig. 1). A statistically significance difference was observed between their basal expression levels in patients who will develop toxicity equal to 0 compared to patients with toxicity equal to 1, showing an up-regulation of their expression levels in patients with toxicity and a difference in toxicity-dependent pattern (Fig. 2).

### Connectivity network analysis between soluble molecules in patients with or without toxicity

**Fig. 3:**
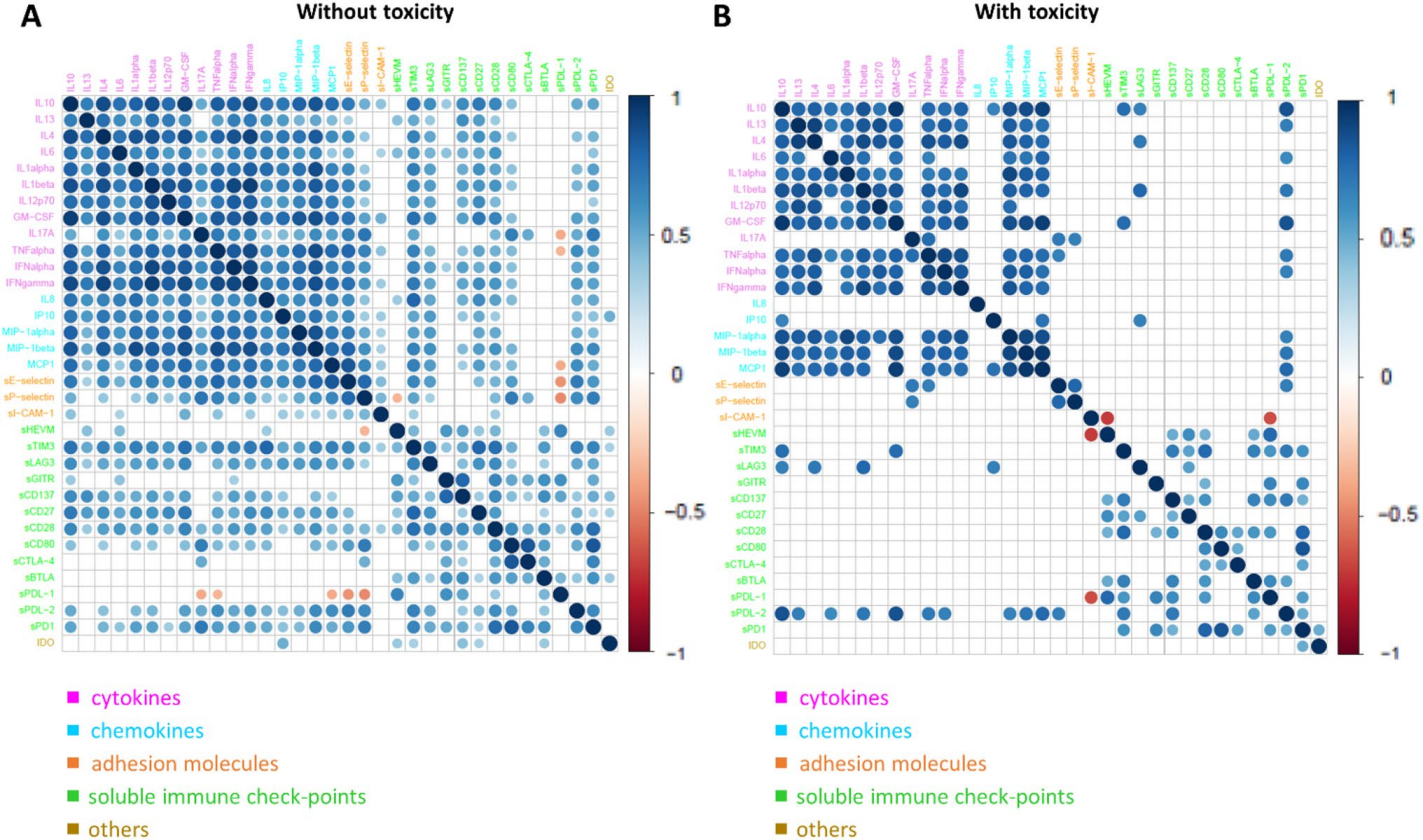
Connectivity heatmap between soluble molecules in patients without toxicity (**A**) and with toxicity (**B**). Statistically significant Spearman correlations (adjusted *p* value ≤ 0.05) are reported. In the plot, circles are scaled and colored according to the correlation values, increasing from red (negative correlation) to blue (positive correlation). Soluble molecules are grouped and ordered according to the functional groups reported in the legend

The connectivity heatmaps between the serum levels of circulating molecules in patients without toxicity (Fig. 3A) and with toxicity (Fig. 3B) provided the image of a markedly different connectivity pattern between soluble molecules, depending on the absence or presence of toxicity.

Patients who experienced a significant increased incidence of irAEs have mostly of the pair-wise connectivity among cytokines disrupt (e.g., most of the pro-inflammatory cytokine connections including IL17A, most of the pro-inflammatory chemokine connections including IL8, or all the soluble immuno-checkpoint connections including sCD137, sCD27 and sCD28), whereas other connectivity pair values seemed to be intensified (e.g., the connections of sPDL-2 with the other cytokines) (Fig. 3).

**Fig. 4:**
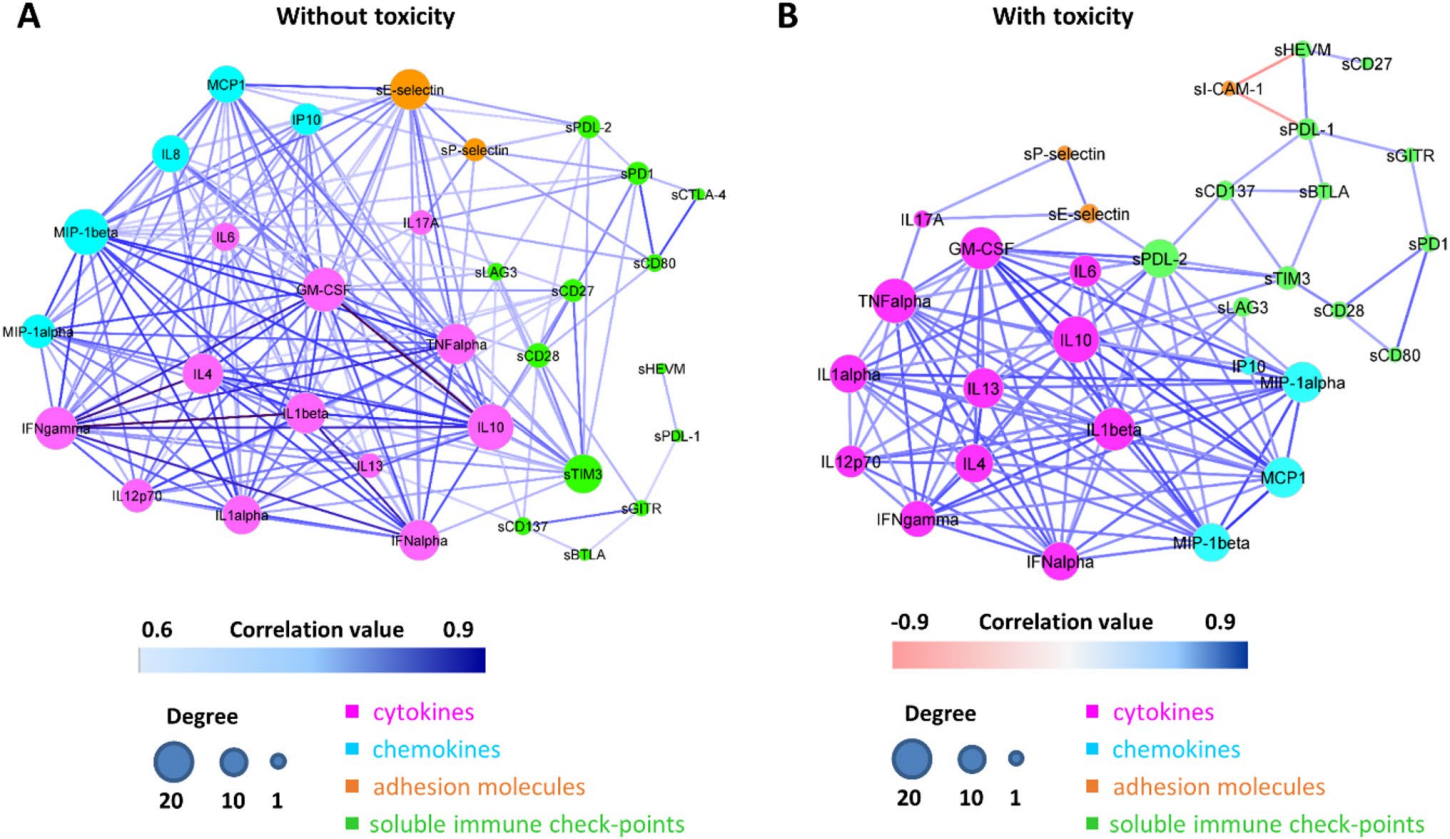
Connectivity network between soluble molecules in patients without toxicity (**A**) and in patients with toxicity (**B**). In each network, nodes represent soluble molecules and a link occurs between two nodes if the absolute value of Spearman correlation between their expression levels is statistically significant (adjusted *p* value ≤ 0.05) and greater than a selected threshold (i.e., the 85th percentile of the overall distribution corresponding to 0.6). Nodes are colored according to the functional groups reported in the legend and their size scales with the network degree (i.e., number of connections of each node), while edge color indicates positive (blue) or negative (red) correlation values

Network connectivity analysis among all possible pairs of biomarkers identified a total of 187 statistically significant interactions in patients without toxicity (Fig. 4A and Supplementary Table 2, first sheet) and a total of 126 interactions in patients with toxicity (Fig. 4B and Supplementary Table 2, second sheet).

**Fig. 5:**
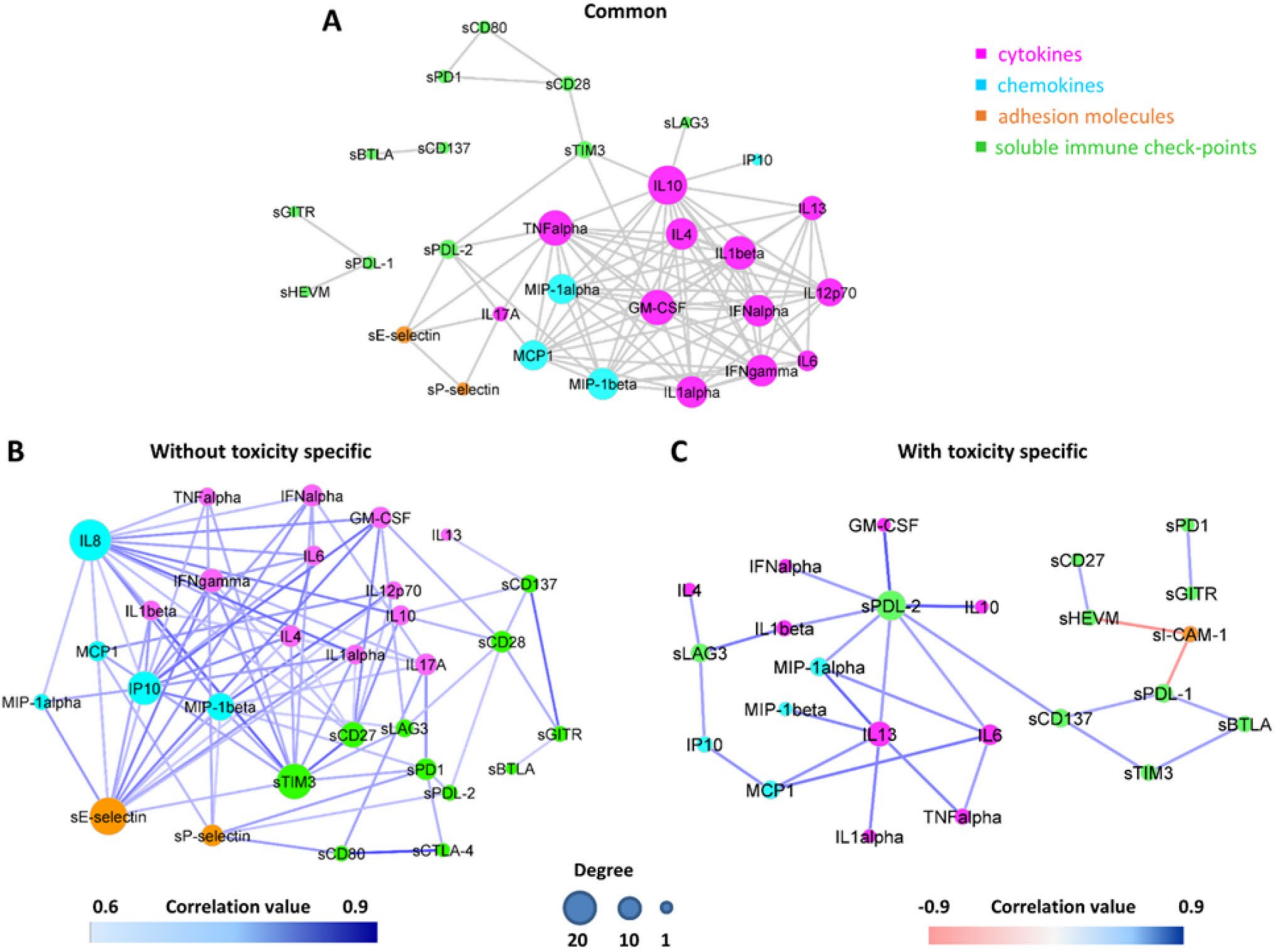
Connectivity network of soluble molecules connections shared between patients with and without toxicity (**A**), specifically present in patients without toxicity (**B**) and specifically present in patients with toxicity (**C**). Networks that are specific for patients without (**B**) or with toxicity (**C**) are obtained by keeping only the connections that are not shared between the two groups. In each network, nodes represent soluble molecules and a link occurs between two nodes if the absolute value of Spearman correlation between their expression levels is statistically significant (adjusted *p* value *p* ≤ 0.05) and greater than a selected threshold (i.e., the 85th percentile of the overall distribution corresponding to 0.6). Nodes are colored according to the functional groups reported in the legend and their size scales with the network degree (i.e., number of connections of each node). In the specific networks (**B-C**), edge colors indicate positive (blue) or negative (red) correlation values

Ninety-eight interactions were common to both networks (Fig. 5A and Supplementary Table 2, third sheet), while 89 and 29 were specifically observed in patients without (Fig. 5B, and Supplementary Table 2, fourth sheet) and with toxicity (Fig. 5C and Supplementary Table 2, fifth sheet), respectively. All these findings pointed out to a clearly distinct signature in terms of network connectivity of the soluble molecules characterizing patients with toxicity.

## Discussion

ICIs represent a breakthrough in oncology and their introduction as a pivotal cancer treatment has notably improved patient survival, demonstrating a favorable safety profile. However, in most cases patients do not benefit and develop irAEs [51]. Our study highlights a correlation between the onset of irAEs and particular expression patterns of cytokines, chemokines, sICs, and adhesion molecules, which suggests a common pattern of immune dysregulation interfering with self-tolerance mechanisms able to promoting or inducing autoimmunity.

Patterns of incidence and side effect severity vary widely in relation to the immunotherapy agent and treatment schedule (combination therapy vs. monotherapy) [52]. An important meta-analysis highlighted that the overall incidence of irAEs related to anti-PD-1/PD-L1 treatment of all grades and G3 was 66% and 14%, respectively [20]. In addition, it was shown that anti-PD-1 treatment showed a higher average incidence of side effects equal or higher to G3 than anti-PD-L1-based therapy [53]. Treatment with ICIs may also stimulate a massive cytokine release syndrome, leading to life-threatening side effects; in selected cases severe or fatal side effects may occur [54].

According to the literature, the toxicity profile in this series was heterogeneous and predominantly of low/moderate grade. The most frequent adverse events involved the skin and the endocrine system. Conversely, gastrointestinal and respiratory system toxicities were rare. One case (1%) of immune-related interstitial pneumonia was recorded. This rare irAE is one of the most serious complications along with cardiac toxicity ones and needs to be promptly and properly treated. Patients rarely (5%) reported neurological symptoms such as headache and dizziness. In addition, the nonspecific symptoms such as asthenia were frequent (33%). Asthenia is related to multifactorial etiology, rarely correlated with true autoimmune toxicities of ICIs. Therefore, in our cohort of patients, affected by NSCLC, UM, R/M HNSCC and RCC, immune-related adverse events are observed in a substantial patient population.

While high-grade irAEs were relatively rare, cumulative toxicity was high. The effect of multiple adverse events, even of a low degree, is a relevant data as they cause a negative impact on patient quality of life (QoL).

A predictive biomarker profile of irAE represents an urgent yet unmet need to better treat patients by preventing unwanted side effects in patients with cancer, avoiding delays or interruptions in immunotherapy and finally improving patients’ QoL.

The high variability in incidence, type and severity of irAEs may be related to the interindividual immune system variability of each patient. The interindividual variability and the fitness of the immunological framework could influence the individual response to immunotherapy treatment and the onset of autoimmune and inflammatory disorders.

Host and environmental factors can affect the immune system, making individuals more or less prone to infectious diseases and cancer at different times. Two large-scale studies confirmed that interindividual variability in immune responses is largely due to differences in sex and age [55, 56] Authors identified several factors which regulate and shape the interindividual diversity of the human immune system that could affect the different responses between individuals to several conditions such as allergies, autoimmune diseases such as type 1 diabetes, cancer and infectious diseases, along with their course.

Circulating adhesion molecules and soluble immuno-checkpoints, as well as cytokines and chemokines detected in patient’s serum, represent promising tools in order to identify patients at risk of irAEs development, even though the huge amount of available data could not be easily interpreted by standard methods.

A positive and significant correlation was found between basal circulating levels of IL8 and IP10, sICAM-1, sLAG3, sPDL-2, sHVEM, sCD137, sCD27 and the development of toxicity in patients undergoing immunotherapy. Interestingly, all these soluble molecules showed an elevated concentration in serum of at baseline, strongly suggesting a key role in autoimmunity, leading to consider a particular immune pattern related to the onset of irAEs (supplementary Table 1). Chemokine IL8’s activates, predominantly but not exclusively, neutrophils inducing chemotaxis, recruitment, powered phagocytosis, neutrophil exocytosis and the respiratory burst [57]. IL-8 is believed to play a role in the pathogenesis of various autoimmune diseases [58–60].

IP10 has been involved in the pathogenesis of multiple sclerosis [61], diabetes mellitus type 1 [62], Graves’ disease [63, 64], autoimmune thyroiditis [65, 66], pulmonary fibrosis [67–69] and cardiovascular diseases such as atherosclerosis [70, 71] and coronary syndromes [72]. Elevated serum levels of sICAM-1occur in many pathologies and are associated with disease progression and severity in immune syndromes, diseases involving chronic inflammation, cancer and cardiovascular disease [73–77]. The 5 soluble immune checkpoints, key regulators of the immune system, positively associated with toxicities play an important role in immune tolerance and autoimmunity [78, 79] (supplementary Table 1).

The immune system is highly differentiated and composed of several interconnected molecules. Therefore, it seems more promising to analyze the role of an immune profile rather than single molecule. Furthermore, each individual molecule could present several dynamic positive or negative interactions in a network immunological context. Each individual molecule has multiple molecules with which it can interact, a bidirectional signaling is possible and, depending on the context, a single molecule could be pro-inflammatory or anti-inflammatory [80].

In this scenario, a novel approach, such as network analysis, appears to be useful in order to understand the interaction between the different circulating molecules and to define some particular profile of toxicity. Connectivity network analysis between molecules provides a markedly different connectivity pattern depending on either the absence or the presence of toxicity. Twenty-nine statistically significant interactions were specifically observed in patients with toxicity. Two distinct soluble profiles were identified. The first, related to patients without toxicity, was characterized by a greater number of correlations between molecules, with connections which also include molecules not belonging to the cytokine/chemokine group, such as sCD137 and sCD27. The second, related to patients with toxicity, was instead characterized by a strong correlation between pro-inflammatory cytokines and molecules not belonging to this class, such as sPDL-2 and MCP1. Patients who experienced a significant increased incidence of irAEs missed most of the paired cytokine connectivity, while other paired connection values seemed to be intensified. There were also some shared correlations between the two groups, such as those involving IL10, IL13, IL6, IL1*α*, IL1*β*, GMCSF, TNF*α*, IFN*α* and *γ*. The molecules showing the greatest correlation in patients without toxicity were IL10, MIP1 and GMCSF, while the molecules with the greatest correlation in the group of patients with toxicity were IL10, GMCSF and TNF*α* (supplementary Table 1).

The molecules with the greatest group-specific correlation which emerged from this study were IL8 for the group of patients without toxicity and sPD-L2 for the group of patients with toxicity. SPD-L2 has been seldom studied, and its role remains partly unclear. Nevertheless, it has been illustrated that PD-L2 is involved in activation of T cells through the co-stimulatory receptor RGMb47 [81]. This mechanism could contribute to the triggering of an abnormal immune response and consequently increasing the incidence of some irAEs, such as atopic dermatitis. Skin inflammation could also be due to PD-L2-related Th2-type activation [82]. In addition, a recent study highlighted that plasma concentrations of sPD-L2 were significantly increased in patients with newly recognized IgG4-related disease, suggesting a potential role in the etiopathogenesis of autoimmune diseases [83].

The main limitation of this study is due to the small sample of patients involved. However, it still provides important insights which need to be further investigated in a larger patient population. Specifically, it would be of great interest to confirm this immune dysregulation among circulating molecules in patients with irAEs, which features the loss of most of the paired cytokine connections.

In conclusion, these results allowed to define, in patients who will develop irAEs, a particular pattern of immune dysregulation. The connectivity network analysis showed that a poorly modulated immune system could ultimately affect immune tolerance and the onset of irAEs. The identification of a basal immune serological profile, predicting the risk of developing immune-related toxicities, represents a new challenge for precision medicine in order to design a customized therapeutic strategy for each patient as to prevent, monitor and treat irAEs.

### Supplementary Information

Below is the link to the electronic supplementary material.

Supplementary file1 (DOCX 18 KB)

Supplementary file2 (XLSX 38 KB)

Supplementary file3 (DOCX 14 KB)

## Data Availability

The original contributions presented in the study are included in the article/Supplementary Material. Further inquiries can be directed to the corresponding author.

## Abbreviations

CT/PET: Computed tomography/positron emission tomography
CT: Computed tomography
ECOG PS: Eastern Cooperative Oncology Group Performance Status
FDR: False discovery rate
HPLC–MS/MS method: High-performance liquid chromatography-mass spectrometry method
GM-CSF: Granulocyte macrophage colony-stimulating factor
ICIs: Immune checkpoint inhibitors
irAEs: Immune-related adverse events
IP: Immune profile
IDO: Indoleamine 2,3-dioxygenase
IP10: Interferon gamma inducible protein 10
IL8: Interleukin 8
IFN-*α*2: Interferon (IFN)-alpha 2
IL-12p70: Interleukin12p70
IL-1: Interleukin 1, IL-1 Beta
IL-1RA: Interleukin 1 receptor antagonist
IL-2: Interleukin
IL-13: Interleukin 13
IL-6: Interleukin-6
IL-17A: Interleukin-17A
MRI: Magnetic resonance imaging
NSCLC: Non-small cell lung cancer
QoL: Quality of life
R/M HNSCC: Recurrent/metastatic squamous cell carcinoma of the head and neck
RCC: Renal cell carcinoma
sLAG3: Soluble lymphocyte Activating 3
sPD-L2: Programmed cell death 1 ligand 2
sHVEM: Soluble herpesvirus entry mediator
sCD137: Cluster of differentiation 137
sCD27: Soluble cluster of differentiation 27
sICAM-1: Intercellular adhesion molecule 1
sCD28: Soluble cluster of differentiation 28
sCTLA4: Soluble cytotoxic T-lymphocyte antigen 4
TNF*α*: Tumor necrosis factor alpha
UM: Uveal metastatic melanoma
