# Supplementary material for: Immune-related toxicity and soluble profile in patients affected by solid tumors: a network approach
Source: Cancer Immunol Immunother. 2023 Mar 3;72(7):2217–31. doi: 10.1007/s00262-023-03384-9 (PMC10264536; doi:10.1007/s00262-023-03384-9)
Supplement: Supplementary file 1 — Supplementary file1 (DOCX 18 KB) [file 262_2023_3384_MOESM1_ESM.docx]

**Supplementary Table 1. Soluble immune molecules correlated with irAEs: characteristics, function and role in autoimmunity.**

| **Soluble molecules** | **Class of molecules** | **Cell source** | **Ligands** | **Main function** | **Role in autoimmunity** |
| --- | --- | --- | --- | --- | --- |
| **sCD137** | sIC | PBMCs | CD137L | - Inhibits CD137/CD137L binding  - suppression of T cell proliferation | Possible involvement in T cell-mediated autoimmune disease |
| **sPDL2** | sIC | Tumor exosomes, alternatively activated macrophages | PD1 | Unknown | Unknown |
| **sLAG3** | sIC | Activated and exhausted CD4^+^ , CD8^+^  T cells, regulatory T cells | Unknown | unknown | Unknown |
| **sCD27** | sIC | Activated lymphocytes | CD70 | transmit co-stimulatory signals and induce activation and proliferation of T and B lymphocytes | It could enhance continuous immune activation in autoimmune disease and chronic infectious disease |
| **sHVEM** | sIC | T cells, B cells, natural killer cells, monocytes, neutrophils and DC | BTLA  LIGHT (TNFSF14) | Unknown | Unknown, but sHVEM levels were found to bee elevated in sera of patients with autoimmune disease |
| **sICAM** | Molecoles of adhesion | B and T lymphocytes  Endothelial cells | LFA-1 | - binding the transmembrane receptor, antagonises leukocyte recruitment  -migration of (activated) leukocytes to sites of inflammation | Unknown, but sICAM circulates at increased concentrations in serum and cerebrospinal fluid of patients with active Multiple Sclerosis |
| **IP10** | chemokine | Monocytes  Endotelial cells  fibroblast | CXCR3 | Leucocytes recruitment | Involved in pathogenesis of many autoimmune diseases, such as type 1 diabetes, Graves' disease and ophthalmopathy, systemic lupus erythematosus, mixed cryoglobulinemia, Sjogren syndrome, or systemic sclerosis |
| **IL8** | Chemokine | Macrophages  Endothelial cells  Platelets | CXCR1  CXCR2 | - chemotaxis  -powers phagocytosis  -ability to mediate infiltration of MDSCs into the tumour environment | involved in the onset and self-sustaining nature of several autoimmune diseases |
| **MCP1** | chemokine | macrophage monocytes | CCR2  CCR4 | -leucocyte recruitment | mediate the recruitment of macrophages and T cells in the Lupus nephritis  - involved in dysregulation of angiogenic homeostasis in systemic sclerosis  - high serum level in longlasting post vaccination macrophagic myofascitis |
| **IL10** | Cytokine | Macrophages  Treg cells  B cells  Mast cells Th2 Tcells | IL10Rα  IL10Rβ | -downregulation of Th1 cytokines  -inhibits CD4 T cell activity  -suppresses expression of costimulatory molecules  -increases survival of B lymphocytes  -blocks secretion of proinflammatory cytokines | Dysregulation of IL 10 producing lymphocites is involved in many kind of autoimmune disease |
| **GM CSF** | Cytokine | activated T and B cells, monocytes/macrophage, endothelial cells, fibroblasts | GM-CSF receptor | - proliferation of granulocytes and macrophages from bone marrow precursor cells  - induces the polarization of the M1-like macrophage phenotype and exacerbates the positive feedback loop of Th17 and Th1/17 differentiation | critical roles in the development of autoimmune Th17 driven diseases |
| **TNF****α** | Cytokine | Macrophages  NK  T cells | TNFR1  TNFR2 | -pro-inflammatory activity  -stimulates cell proliferation and survival  -induction of apoptosis  -implicated in resistance to antiPD1 drugs | -inappropriate or excessive activation of TNF-α signaling is associated with chronic inflammation and autoimmune disease  -central role in reumatoid and psoriasic arthritis, iflammatory bowel disease, uveitis |

s- soluble; CD137: cluster of differentiation 137; PD-L2: Programmed cell death 1 ligand 2; LAG3: lymphocyte Activating 3; CD 27: CD27: cluster of differentiation; HVEM: Herpesvirus entry mediator; DC dendritic cells; ICAM-1: Intercellular Adhesion Molecule 1; IP10: Interferon gamma inducible protein 10; IL8: interleukin 8; MCP1: Monocyte chemoattractant protein-1; IL10: Interleukin-10; GM-CSF: Granulocyte macrophage colony-stimulating factor; TNFα: tumor necrosis factor alpha
