# Supplementary material for: Immune-related toxicity and soluble profile in patients affected by solid tumors: a network approach
Source: Cancer Immunol Immunother. 2023 Mar 3;72(7):2217–31. doi: 10.1007/s00262-023-03384-9 (PMC10264536; doi:10.1007/s00262-023-03384-9)
Supplement: Supplementary file 3 — Supplementary file3 (DOCX 14 KB) [file 262_2023_3384_MOESM3_ESM.docx]

**Supplementary Table 1.** This table is composed by “Soluble immune molecules correlated with irAEs: characteristics, function and role in autoimmunity”

**Supplementary Table 2.xlsx** This table is composed of five sheets reporting all the connectivity networks as edge-lists along with the correlation values and their corresponding adjusted *p*-values. The first ssiheet lists the soluble molecule network connections for patients without toxicity (depicted in Fig. 4A); the second sheet lists the soluble molecule network connections for patients with toxicity (depicted in Fig. 4B); the third sheet lists the soluble molecule network connections shared between patients with and without toxicity (depicted in Fig. 5A); the fourth sheet lists the soluble molecule network connections specifically found in patients without toxicity (depicted in Fig. 5B); the fifth sheet lists the soluble molecule network connections specifically found in patients with toxicity (depicted in Figure 5C).
